# Understanding and Self-Organization

**DOI:** 10.3389/fnsys.2017.00008

**Published:** 2017-03-02

**Authors:** Natika W. Newton

**Affiliations:** Department of Philosophy, Nassau Community College, State University of New York (SUNY)Garden City, NY, USA

**Keywords:** understanding, self-organization, consciousness, representation, recursion, emergence

## Abstract

How do we manage to understand a completely novel state of affairs, such as the sudden effects of an unexpected earthquake, or the arrival of a total stranger instead of the sister we were waiting for? In each case, for a moment we might be stunned, but we are able quite quickly to fit these events into our overall framework for understanding the world. However, terrified and despairing we feel, we know what earthquakes are and this event fits that schema; in the case of the stranger we know that this kind of thing happens, and that we must ask the stranger “Who are you, and where is my sister?” This paper asks about the mechanisms by which we rapidly achieve an understanding of our world, both the unexpected changes we may experience, and the ongoing comfortable familiarity we normally have with our surroundings. We attempt a solution by means of examining fundamental questions:

- What is it to understand something?
- What sorts of things do we try to understand?
- Is there a conscious EXPERIENCE of understanding?
- Does understanding involve conscious mental images?
- What is self-organization?

I will argue that these questions revolve around the need of a living organism to take action, and that understanding anything involves knowing how we might act relative to that thing in our environment. The experience of understanding is a feeling that the action affordances of a situation are clear and available. Action (as opposed to reaction) includes imagery, particularly motor imagery, which can be used in the guidance of action. Understanding requires a conscious process involving motor imagery of action affordances, and action can be understood only in self-organizational terms. I explain how self-organization can ground the kinds of action affordance experience needed for conscious understanding. The paper concludes that our day-to-day understanding of our environment is the result of a self-organizing process.

## What is it to understand something?

Answering this question requires distinguishing two ways it can be taken. One way is as a “success” term, with the assumption that there can be correct and incorrect understanding. Thus, we can say of someone that she thinks she understands the term “water” if she views water as any clear liquid that quenches thirst, but that she does not really understand it, being ignorant of the molecular composition of water. The other way of taking the concept of understanding is purely as a mental state of a subject, in which she finds for herself a way of interpreting something (word, sentence, object, or event) that allows her to use her interpretation to think about or act upon her interpretation in a way that satisfies her. We can call this “having AN understanding” of something. Having an understanding in this sense does not involve success or failure, as long as the subject herself is satisfied.

In this paper we are concerned with the second sense of understanding. The former sense is the subject of philosophy of language, while the latter sense concerns only what is going on in the subject's head—i.e., her nervous system. Reaching a state of having an understanding is independent of “correct” definitions, or of knowing the correct meaning in a given language, and is concerned solely with the subject's experience. While the interpretation does not necessarily map onto the objective world, it allows the subject to use her interpretation to incorporate it into existing schemas and to create new models consistent with it. If what she understands in this way is incompatible with the objective world, she will sooner or later discover this, and will have to revise her understanding or abandon it for one more compatible in objective terms. She still has an understanding of the object or situation, which is again not necessarily accurate, but which allows her to act on this revised interpretation.

For example, suppose Susan is rude to her friend Tom, who consequently is hurt and interprets Susan's behavior as evidence that she no longer likes him. His understanding of her behavior satisfies him intellectually, although of course he is disturbed, and his interpretation allows him to decide to snub her at their next encounter. But when he does so she bursts into tears, while his friends tell him that she has been under a great strain and is seriously depressed. Then Tom will no longer interpret her rudeness as a sigh of rejection of him personally. Once again, his new interpretation may be objectively inaccurate, but it provides *an* understanding that allows him to interact with Susan in a way that is intellectually comfortable.

Finally, we can be even more precise by looking at a clear *lack* of understanding, in the well-known example of the Chinese Room (Searle, 1984, discussed at greater length below). Searle imagines himself going through all the motions of a computer with a translation function, which receives questions in Chinese and delivers answers, still in Chinese. Someone on the outside might well-believe that the computer understands Chinese. But Searle, as the computer inside the room, knows that he does not understand, and we can certainly take his word for it.

## What sorts of things do we understand?

Most commonly, we think of understanding *language*. But in fact we must have an understanding of every aspect of our lives—every object we encounter, every event we are involved in or witness, everything that is part of our environment. Without this understanding, we are at a loss as to what to do next. It may sound strange to speak of understanding an *object*. But in the case of any object we need to know what it is for, how we may use it or avoid it, what actions it *affords*. Even a meaningless rock lying in a field can be picked up, thrown, taken home as an ornament, etc. We might be mistaken in our particular understanding of an object if we think a rock is a mushroom and try to bite into it; in that sort of situation we search for a different, we may say more successful understanding. Leaving some aspect of our environment not understood is a worry; we have to figure it out so as to know what to do with it or expect from it. We seek an understanding of events, such as two people whispering at the faculty meeting. What can they be whispering about? We are then able to interpret their whispering as an attempt to locate the memo referred to by the speaker, and we then can go on to the next issue in our general attempt to understanding the meeting as a whole. The unspoken premise here is that being alive is for us a process in which we are always acting in relation to our environment, however minimally (Ellis and Newton, 2010). Lack of understanding obstructs the process of acting, and hence is felt as a problem.

## Is there a conscious experience of understanding?

There must be conscious experience, at least in the initial encounters with a thing, because having an understanding puts us at ease, enabling us to feel that we can make use of what is understood, or incorporate it into our global experience. In other words, the above is *what it is like* to have an understanding. Lacking any understanding leads to puzzlement and a feeling of insecurity or discomfort: what are we supposed to do in this situation? If we are in an apathetic state because of depression or if we are sufficiently distracted by something else, we may not try to understand (This experience can also be common when we do not care whether we understand or not, as during a murky movie when we have given up and stay only because of politeness). But if we are called upon to DO something about the thing, or perform some actions in light of it, we must attempt to arrive at an understanding, to incorporate the thing into the rest of our current situation.

The discomfort of lacking an understanding—“What's going on?”—leads to attempts to arrive at an understanding, and when we are successful there is the well-known “Ah-ha!” experience. This is normally a positive experience—the discomfort is eased—unless understanding reveals the object to be scary, dangerous or sad. Even then, we are better off for knowing how to react. As we grow accustomed to our familiar environment we take the understanding for granted; if someone brings me coffee while I am working I need not go through a moment of puzzlement, unless this offer of coffee is not at all typical of the normal behavior of this person. Then I might ask some questions. The upshot is that normally, when we have an understanding, we are comfortable in our surroundings, without much thought, and we are unpleasantly aware when we lack it. So we may speak of the “A-hah!” experience, and the “What's going on?” experience, as *what it is like* to have or lack an understanding.

It should be clearly noted that we have access to our state of understanding—we may say “privileged access”—we know when we understand something and when we do not. It can be argued that we have no special access to our mental states, which are determined in part by the environment. But this objection applies to the first sense of understanding discussed above, which we are not concerned with in this paper. Here, having *an* understanding is a state of which the subject is fully aware. “Accuracy” of understanding does not apply. In some situations I might pretend to understand what's going on, when I really do not. No one else may notice, but I know the difference. This situation can lead to social complexities and confusion, but I myself am usually aware of my role in the awkwardness.

John Searle's “The Chinese Room” is rich in examples. Searle argues that the computational theory of mind—the theory that thinking is manipulating symbols, meaningless themselves, that we have learned correspond to objects and events in the environment—is not accurate. His main argument, that syntax does not yield semantics, uses the well-known example of a person inside a closed room, manipulating Chinese symbols in response to input, matching input with output by following rules in a book. According to computationalism, Searle says, correct manipulation—syntax—should be equivalent to knowing Chinese. But in that case, Searle said, the person handling the symbols should understand Chinese. Searle, imagining himself as the person in the room KNOWS that he does not understand Chinese. So the computational theory is false.

Not only does he know that he doesn't understand Chinese, he knows how to read and obey the instructions in the English manual. His knowledge is made clear, to us and to him, by the fact that he obeys the instructions with no difficulty. To provide a personal anecdote: I was once playing a word game with another person, who for a while seemed to be keeping up with the rules. But she claimed not to understand what she was doing, but was just lucky; when I explained the rules to her, she repeated that she had not understood how the game was played, but that now she did. She clearly described states of not understanding, and of understanding; it seems to follow that those states yielded conscious, reportable experiences (This example is discussed in Newton, 1996).

## Does understanding involve conscious mental imagery?

Mental images have traditionally been thought of as primarily visual—a picture of something is often called an image while a 3-D representation of a structure is a model; an annoying, persistent memory of a tune is “a tune in one's head.” But tunes in one's head are no different from pictures in one's head in their central function: they are imagined reproductions of past audio-visual stimuli that we have experienced and now remember. We can recall flavors—taste images; pains—pain images; extreme temperatures—heat or cold images. There seem to be no sensory experiences that cannot be reproduced as mental images: they are like the actual experiences, but are no longer objectively present (Pearson and Kosslyn, 2015).

We can also have proprioceptive images, images of events in our bodies, such as hunger pangs, or motor images, which reproduce the sensations of moving parts of our bodies. Motor images have received much attention in recent years for their role in generating overt bodily movements (Sacks, 1984). According to Jeannerod (1988), movements of our limbs begin with imagery of the movement in the motor cortex; this imagery is activated by allowed to proceed to the sending of nerve impulses to the muscles, which execute the movement. The action can also be prevented prior execution by inhibitory signals in the cortex. Proponents of free will argue that while arm movements can be predicted prior to execution by activation of the motor image, there is still time for the action to be inhibited at the last minute; during this short time the subject can choose inhibition or not (Libet, 1985, p. 143). Whether or not this account is successful will not be discussed in this short paper.

We have seen that having an understanding of something means knowing how one might use or interact with the object or event to be understood. If, as we have argued, there is a feeling of understanding that is conscious, then this feeling must consist in the experience of representations or imagery of some of the possible interactions. For example, suppose you enter an unfamiliar gym and see a novel type of equipment. You can ask how one uses it, or you can simply look at it and try to figure out where the feet go, where the arms go, what types of motion the equipment allows. You are trying to understand the equipment, and the attempt entails sensorimotor imagery of interacting with the machine, imaging what the motions will feel like to execute, etc. You can do all this while passively observing the machine.

Is there any other way of coming to understand the machine? Suppose you ask the attendant to explain it; can't you understand and apply his verbal information (“you step on the foot pedals and hold on to those bars”) without producing motor images in your head? No; the images let you know if you can do what he says. Don't we do that kind of thing all the time? That question takes us back to the discussion of conscious understanding. It was argued that having an understanding of something means knowing how one might interact with it, use it, participate with it in some way. Often this kind of knowing is non-verbal. For example, you know how to keep your balance on a bicycle while riding it, and you can imagine doing so. But if you are asked to verbalize this knowledge you may be at a loss. When riding, you tighten and flex muscles, and shift your body weight around, in ways that feel automatic, that you can image clearly through proprioceptive and motor imagery. Confronting a bicycle with understanding, we might say, means generating an image of how you would ride it (And if you cannot generate such an image you will feel, or protest, that you don't know how to ride it). Thus of the two apparently possible ways of having an understanding of riding a bicycle, only the way involving imagery will be satisfying to you. Hearing the explanation without knowing what it would be like for you to ride it does not help you understand, if you do not understand the elements of the explanation. And knowing what it would be like for you to ride is being able to generate imagery of your body in the act of riding. Thus, having an understanding of something involves conscious mental imagery. The only exception will be in cases where you have performed the act so often that you feel sure, without mental rehearsal, that you are familiar with the object or event. But even in those cases, your feeling of confidence can be an “image,” in an attenuated sense of image, of traces of the “Aha” feeling that you achieved when you originally developed an understanding.

Try an experiment: suppose you are asked if you are able to reach the vase on top of the bookcase. How do you decide? If you aren't sure, try introspection. Do you not imagine standing in front of the bookcase and reaching up? Perhaps you have a motor image of standing on your toes and straining to touch the vase. If that image leaves you undecided, you can walk over and try to reach the vase, and get the right answer.

It can be objected that if you know quantitatively the height of the bookcase, and your own height with your arm length added, you could find the answer with no imagery necessary. And the objection is correct, in that you can now find the answer by simple addition. But that fact leads to another case of conscious or semi-conscious motor imagery: the dependence of arithmetic on representation of basic action patterns. Let us look in detail at the most abstract way we use action imagery. I have been defending the claim that understanding, in the sense that once has AN understanding of something, is a process that maps novel stimuli or experiences onto an original structure that is already understood. The novel material, thus mapped, is understood as well as the original material, in that it has become part of the subject's repertoire of usable structures, or mental models.

For example, suppose we call an original structure “reach, grasp, pull.” This structure emerged, let us say, when the infant first saw a desirable toy and grabbed it. The structure of that act is the act itself; it is understood because it is created by the infant in response to her own desire. It is the means of satisfying that desire. Now that she has that movement pattern in her repertoire she can use it at will to obtain other desirable things. She is also now ready to use that pattern in other circumstances, to interpret concepts or environmental input that goes beyond immediate satisfaction of a desire for an object. Suppose she is now older and is told she will be taken to a store to buy new shoes. She can understand that prospect easily once she can see it as another instance of “reach, grasp, pull;” going to the store is reaching, selecting, holding, and buying the shoes is grasping, and wearing the shoes home is pulling.

What about the novel qualitative and quantitative properties that are structured by the familiar pattern? How are they understood? It seems correct to say that they are experienced as properties of the fundamental pattern, and are not viewed as distinct from that pattern, but as features of experience that merge with the pattern. If they are purely qualitative, they can be experienced but not described except in terms of a pattern. In describing quantitative properties of the shoes, other recursive patterns will be applied to the details of buying the shoes, the trip, the transaction, etc. E.g., the car is a thing that you get into, and that moves you from point A–B. The red color of the shoes, on the other hand, cannot be described but only experienced and named. Sensory qualities like the smell of the leather, the smoothness, the heft of the shoe are unanalyzable, but as properties of the object are subject to the object's handling and do not need sensorimotor patterns of their own. In general, qualia are presented to us as bound to objects and events we understand through motor imagery, and do not present philosophical difficulties in ordinary circumstances.

We can abandon sensory qualities in purely abstract contexts like mathematics. Now imagine that a child is learning how to add three digit numbers, and must understand what it is to “carry” a numeral. The “reach, grasp, pull” pattern can be applied in an attenuated form here: when we add 123–789, for example, we must “carry” the one from the rightmost column to the middle one, where it is incorporated into the sum of 8 and 2. The term “carry” is clearly metaphorical and derived from physical operations: “add” can be understood in terms of placing one object into a collection of others, “divide” in terms of separating *n* sets of objects from a larger collection, etc. Note that the “reach, grasp, pull” structure is only one of many metaphors based on bodily movements in space: *into* and *out of* are derived from experience with containers in which we can put things or find ourselves inside of; and they can structure metaphors in vastly different contexts—e.g., “the voters put him *in* office”; or “three *goes into* nine three times.”

The basic point here is very simple: we understand our bodies in being able to move them and use them to satisfy our wants and needs. Understanding anything has its roots in this ability. Our bodies are our tools for achieving our goals, and there is nothing more to having an understanding of them than intentionally moving them. Because our essence as agents is expressed in conscious, voluntary bodily activity, then having an understanding of our own voluntary actions is itself nothing more than being intentionally able to engage in bodily activity or imaging it (knowing what it is like). We understand ourselves, moreover, *as* voluntary agents. Being self-aware is being aware of our bodies, not as something we, as disembodied subjects, *have*, but as what we are.

Understanding anything, in the sense we are using it, involves situating it into contexts or structures with which we are already familiar. As a person grows and acquires new experiences, the original sensorimotor structures of early life stretch to accommodate these experiences in ways that he can “make sense” of, in light of the earlier experiential structures. It is hard to imagine how one could develop any understanding of novelties except by connecting them with prior experience. An example from academia is the teaching of Plato's Theory of the Forms. The instructor is helpless to explain what a Form is unless she can find something in students' experience to relate it to. She can try beginning, for example, by explaining that Plato's realm of Forms is to the world of concrete objects as, in Christianity, Heaven is to Earth. The success of this move depends upon the students' understanding of Christianity, and that understanding depends, in turn, in part on spatial metaphors such as “above,” for Heaven, and “below” for Earth. In other words, trying to explain a complex new concept in terms pertaining only to that concept, with no terms from the hearer's experience to ground the explanation, is useless. The hearer can learn which new words to use with which other new words, but, like Searle in the Chinese Room, will have no grounded understanding of the concept that she can use to think about it in a satisfying and possibly creative way.

Yufik and Friston propose a theory of understanding (this issue) that is highly compatible with that of this paper, except that their theory focuses on neuronal events underlying understanding, while this one is more concerned with mental acts on a conscious level. One might say that their theory is more “bottom up,” mine more “top down.” Importantly, both theories exemplify enactivism—the view that cognition is a mode of human activity, and both theories emphasize the role of action representations, or mental models:

Notice the two key themes of this formulation are an emphasis on active inference or volitional sampling of the world—of the sort that characterizes enactivist or situation approaches to cognition. Second, the progressive elaboration of internalized (“as if”) stimulus-response links induces conditional dependencies between the sensory input and internal models of how those predictions were caused—through active sampling (Yufik and Friston, 2016).

In summary: Understanding is tightly coupled with the need of a living organism to take action. Understanding involves knowing how we might perform goal-directed actions relative to the environment. The experience of understanding is a feeling that the action affordances of a situation are not entirely unclear. Action (as opposed to reaction) requires imagery, including motor imagery, that can be used in the guidance of action.

## Self-organization

With this sketch of a theory of understanding, we will attempt to see it as a self-organizing process. To do that requires that we first consider it as a recursive process. Not all cases of recursion are biological, like our theory of understanding. Just as many writers view intentionality as a property of natural language, logic, mathematics, and language provide instances of recursion: the application of a function to its own values to generate an infinite sequence of values. “***Recursion*** occurs when a thing is defined in terms of itself or of its type” (Wikipedia, *Recursion*).

In what follows we examine the concept of self-organization particularly as it takes place in an organism—self-organization as a biological property. The much broader research project originated in applications to dynamical systems theory (Ashby, 1947), followed by physics, chemistry, computer science, and more recently to human behavior. A major influence was the work of I. Prigogine on self-organization in irreversible thermodynamic systems, for which he won the Nobel Prize for Chemistry in 1977 (Prigogine and Nicolis, 1977). Among other central thinkers is Hermann Haken who finds highly important examples of self-organization in brain function (Haken, 2008; Karsenti, 2008).

Below we look at two properties central to self-organization in any type of system, including the brain and human cognitive functions in general.

## Recursion

Language is *recursive* when a type of clause in a sentence is used to make a new sentence. For example “Bobby went to the store” can become a new sentence “Bobby went to the store and to the pharmacy,” and “Bobby went to the store and to the pharmacy, and to the movies.” The prepositional phrase “to the store” is a grammatical function within the sentence, and that function can be infinitely repeated to make new sentences. Mathematics is recursive when a number series *n* is extended by “*n*+1” and “*n*+1 +1.”

Recursion is part of a complete definition of language and mathematics, and the concept has been used to deny language ability to intelligent non-human animals. Recursion also occurs naturally in non-living entities such as crystals (e.g., snowflakes and blocks of quartz), and it frequently results in emergent properties, such as the symmetry of crystals, the shapes of flocks of flying birds, or traffic patterns, which maintain their overall shapes as their sizes change.

Metaphors are essential tools in understanding, both linguistic and otherwise. We understand something by being able to see it in terms of something we already understand. That fact may help to explain our inability to articulate a definition of sensory properties like color; red itself has no articulable components that can be compared to or mapped onto anything else (Lakoff and Johnson, 1987).

The examples of recursion we have been examining include both inanimate, fixed structures like language and mathematics, events like presidential elections, and animate biological processes. We have argued that understanding is a biological process, based on recursive iteration of action structures, or action images. The importance of recursion in our theory of understanding is that, through recursive processes in which structures are extended to new data, the new material is understood simply by being incorporated into a wider context already fully understood.

We are now ready to understand how forming an understanding of elements in our environment involves *emergent properties*.

## Emergent properties

Emergent properties are properties of the patterns resulting from self-organizing processes that are not present in, or predictable from, the individual components that have been organized. A well-known example is traffic patterns: “In the case of a traffic jam, what appears is an entity whose properties need not have anything in common with the properties of its constituent units (cars). In particular, one may have a stationery or even moving back traffic jam while all cars are moving forward. This higher level structure, whose equations of motion are not easily derivable from those of cars, emerges from the interactions between the cars” (Bonabeau et al., 1995). Because the properties are not properties of the individual “agents” but only of the organized whole, these properties are “emergent;” meaning that they did not exist previously in nature, and could not have been predicted from the properties of the “agents.” They are more than, and different from, a mere aggregate of the agents that make it up (Anderson, 2011).

Another important feature of emergent properties is that they have causal powers not found in the individual entities making them up. Traffic patterns, when they cause tie-ups in the traffic flow, cause not only traffic jams but also extreme irritation on the part of the drivers involved. But the traffic jams and the irritation are not caused by individual cars, because even if a single car is driving too slowly for the comfort of other drivers, they are not forced to stay behind it, but can move around it freely, as they could not in the case of a traffic jam (Kerner, 1998). Other properties of collections of individuals, such as electorates that exist at a higher level of organization than the parts, are not in themselves emergent properties, since electoral powers are as true of a mere aggregate of the individuals as of an overall electorate, whose causal properties are reducible to the aggregate of causal powers of individual voters (Ellis, 2012). One might say that the “emergent” causal powers exist because the individuals are arranged in a particular pattern, and that property is true of the individuals as aggregated. It is true that a particular arrangement of individuals has led to the “emergent” properties. These properties, however, are previously unseen in nature, and were not predictable from knowledge of possible aggregates of individuals. They appear, moreover, spontaneously out of chaotic states of individuals, and are not composed by external intelligent agents. Thus, these patterns with their novel properties can be said to emerge from chaotic states because of causal powers of their own.

The concept of emergent properties is now so well-established that it has tempted some to apply the term in cases where the “agents” are not well-understood: for example, consciousness has been called an emergent property in humans and some non-human animals. This would be an appealing way to solve the hard problem of consciousness, if the “agents” that would self-organize to produce it are known. They are presumably states of the brain, but unlike individual cars in the traffic pattern case they have never been observed to create the emergent property of consciousness in which they could be found. Emergent properties are properties of groups of entities that can be observed as part of the final form, not losing their material nature. In the case of consciousness, it seems that no individual entities, or agents, are in any way observable or detectable in conscious experience, which manifests a unity for the conscious agent.

Understanding, however, as we have been using the term, *is* an emergent property of biological processes. The entities that lead to forming an understanding of something are detectable, and can be analyzed out of the experience of understanding. An experience of understanding contains, at least, motor imagery of familiar action patterns, a mental state of puzzlement or tension followed by a relaxing of the mind into the structure and affordances of what is understood, and confidence in the planning of future actions related to the entities or situations now understood. The state of understanding as a whole, moreover, has causal powers (in a given situation) that its components, representations of action patterns, do not have. A satisfying feeling of understanding, such as the “Aha” experience is accompanied by images or representations of action patterns, which the subject uses metaphorically to interpret the novel state of affairs—object or event. These representations may not be the most prominent aspects of the experience, in which attention would be focused on the newly-understood state of affairs. But they are introspectively available. In themselves, these action representations do not constitute understanding of the novel situation, but together with representations of the current stimuli do combine to form a whole scenario that is understood. Without the emergence of understanding, as it is presented here, normal human life would be impossible; one would literally never know what to do. The components of understanding must unite for any functioning in the world to occur.

One source of evidence for the role of action representations is the work of McNeill (1992), who studies the role of gesture in expressing such representations:

For example, consider a speaker who says, “I was holding a big box” and produces a gesture that mimes holding a big box. In this case, both modalities express the same idea, so the degree of redundancy between gesture and speech is high. The gesture also expresses additional nuances of meaning, such as information about the position of the hands as they hold the box, but the semantic information expressed in the two modalities is largely overlapping. At the other end of the continuum are cases in which there is little or no semantic overlap between the two modalities. In one often-cited example, a speaker describing a scene from a Sylvester and Tweety cartoon said, “she chases him out again” while swinging her arm as if wielding a weapon. In fact, the speaker was describing a scene in which Granny chases Sylvester while swinging an umbrella. In this example, the speaker expresses an aspect of the scene in gesture (swinging the umbrella) that she does not express at all in speech. Thus, in this case, the degree of redundancy between gesture and speech is low (Alibalia et al., 2009).

On this account the speakers are expressing in gestures the motor imagery they are using to describe a scene. The use of gestures indicate that the speaker is thinking of action patterns that she uses to understand and describe the scene. In the second example, the speaker is thinking of a component of the scene that she does not express in words, indicating that she understands the scene herself in terms of her prior experience of swinging an object in her hand.

To summarize this section: understanding is a state of mind that emerges from the blending of other mental states, driven ultimately by the emotion of wanting to be comfortable in one's environment. The mental states of experiencing motor imagery of action patterns and sensory input from the environment, and relating these metaphorically to basic action patterns learned by experience in infancy, create an emergent state of confident action planning which would be impossible if these components were not united by the emotional drive to be “at home” in the world.

## Self-organized understanding

What elements are self-organized in the case of understanding? The basic constituents are the simple bodily movements themselves. After mastering the reach-grasp-pull pattern, an infant soon finds that the pattern can be extended in various ways, such as grasping two small things at once between reach and pull. We can say that the larger pattern (reach-grasp-grasp-pull) is self-organized if a) the change was not conceived by the infant in advance, but was a spontaneous extra-grasp addition to the original pattern—in other words, the repeated grasp creates a higher order pattern reach-GRASP-pull, with the intervening GRASP now encompassing two smaller iterations.; and b) the process is recursive, in that components of the original pattern are used to construct an emergent pattern within the same structure. We assume here that the infant is not thinking out this plan in advance, but is responding to a motivation—to obtain the toys—in a somewhat automatic way.

Let us look at higher-level processes of understanding. Take Searle's example of a person in the Chinese Room. His instructions are to take the input, consisting of Chinese characters, look them up in a book, and return the prescribed different set of characters through the output slot. Certainly he doesn't understand the characters. But he does have a clear understanding of what he is supposed to do. Not only can he express them in English (as he does in his article), he understands them in terms of our basic pattern of reach-grasp-pull. He reaches for them as they come in the input slot, grasps them, and pulls them to him and looks at them. This case of understanding is, of course, very simple, and probably minimally conscious. The notion that reach-grasp-pull clearly applies here, and can be clearly extended to apply to any abstract cognitive tasks in the “grasp” mode.

I have described the application of pre-learned sensorimotor patterns to examples of simple tasks to make clear the recursive activities involved in a range of cases of emergent understanding. One more aspect of understanding, seen as a recursive activity built upon basic patterns, is the motivation that leads the understanding subject to apply the patterns, with growing sophistication, to the constantly arriving new states of affairs that must be incorporated into the subject's world view. How do we know to keep applying the same basic patterns to novel input? We need a concept to express the growing facility with which we incorporate novel states of affairs into our world-view. Why do we not struggle for understanding in the case of radically novel input, not to mention the constantly changing environment with which we are confronted moment by moment? Not only is there normally no struggle, but the basis for understanding a novel state of affairs is in place *before* we can puzzle over the scene. The general schema for understanding our world allows an even flow from one scene to another, seamlessly, because we are motivated to “look for” such a framework before the event of new sensory input.

The recursive building activity that lets us feel at home in the familiar but changing world is known as *stigmergy*. As Camazine et al. (2001) explains, referring to stigmergy:

[a] process of decentralized coordination …where individuals respond to stimuli provided by the emerging structure itself can be a rich source of information for the individual. In other words, information from the local environment and work-in-progress can guide [and motivate through positive feedback] further activity. As a structure such as a termite mound develops, the state of the building process continually provide[s] new information for the builders (p. 23).

The preceding quotation applied in the original to termites, but it can apply equally well to cognitive understanding in an intelligent individual. As we grow in the world, novel conditions inspire more use of sensorimotor patterns to understand them. Like a growing termite mound, our growing understanding of the world, via *stigmergy*, supplies a conscious sense of satisfaction and guards against confusion at first encountering the new situation. The individual termites have innate instructions that lead each one to coordinate its activities with the others, while being unaware of the activities of the others and focusing on its own tasks. In the case of us humans, we need more conscious motivators; our pattern-use is not a result of blind innate drives. Our motivators, as mentioned earlier, are conscious feelings of satisfaction and discomfort. We are normally uncomfortable when confused, and seek understanding. When we have achieved that, we are satisfied; our work at building our extended world is completed. We seek to be *at home* in the world, not at a loss. When at home, we know what we can do next. We can simply say that the more we understand the better we feel.

So far I have discussed the use of sensorimotor patterns to incorporate novel situations into a given cognitive framework. Sometimes, more rarely, there appear cases of true novelty, structure-breaking events that require almost complete re-evaluation in terms of the subject's previous system of understanding. All of a sudden all the lights go out. It is pitch-dark outside my open window, and surrounding me inside. Nothing has prepared me for this. It is as though I have gone blind in an instant. There are no clues as to what I should, or even can, do. What next?

My own physical body is my sole remaining anchor. I can take stock of where my limbs are and what objects I can touch. Two new interpretations are available to me: I am totally blind, or something has happened to the light sources outside me. The latter seems a more hopeful option; I try to construct plausible scenarios, and finally find one involving unnoticed growing lateness of the hour (the outside darkness), and a major electrical fuse blowing (the inside darkness). That works.

In true novelty, when I would not have even my body to anchor me, there might be no plausible scenario. In such an extreme case, with no familiar structures to turn to, I could only search my cognitive repertoire for some logically possible explanation, in terms of what I am acquainted with. If that search failed, as it would with no proprioceptive input whatever, I can suppose that only blind panic would take over. That state is unimaginable to a normally embodied subject (like this author). A brave attempt to imagine it can be found in the novel “Zero K” (DeLillo, 2016, p.155ff) when a newly disembodied subject first awakens to her situation). The conclusion I draw is that the body is foundational for any self-aware cognition with which to construct some degree of understanding. With the body I can tell some sort of story; without the body, there is nothing—even memories of embodiment would not locate *me now*. Truly novel situations are possible only against a background of minimal familiarity; take that away, and subjective cognitive activity must cease. But with some element of familiarity, which must include some degree of embodiment, a possible world might be constructed to fit my experience. Understanding requires embodiment, and thus any understanding will be structured by actual or possible bodily actions (Boden, 1990).

Returning to the subject of self-organization, one might argue that the preceding is not an explanation of self-organization, since we, the subjects, consciously monitor the construction of understanding, which means that the successive states of applying motor patterns to our environment are not independent of “intervention by external directing influences.” And it is true that the subject of the understanding is consciously trying to understand, motivated by a need, and that she therefore accepts or rejects candidate patterns with which to organize the new data. Nevertheless, she selects or rejects by means of her conscious feelings only, and not by an independent standard of fitness of the pattern with what has gone before. She need not know that the successful pattern is a metaphor for the basic patterns of movement that she has carried with her from infancy (and as I have discovered, may well deny it when it is suggested to her!). Here is a personal example: I was trying to convince a colleague that his memory of a bad experience at a party was structured by a sensorimotor pattern from earlier experiences, and that his memory of the party was composed of representations of those experiences, and not of purely linguistic representations such as “I had a bad time at the party.” He denied it, so I asked “when you think of the party, is there any bodily reaction that you are aware of?” and he answered “I wince.” That convinced him (at least partially) that such memories are not independent of personal patterns of reactions to unpleasantness.

The point is that the unpleasant experiences used to structure the memory of the party, and motivate the wincing, were in place before my colleague reacted to them. The feelings of satisfaction that arise when we find a pattern with which to interpret anything new are automatic, and we can then proceed to use our understanding with confidence, knowing that we have successfully expanded our experienced world. And, as we see with the example of sudden, complete sensory deprivation, motivation to construct a pattern would be baseless, and could not begin. To be a self is, necessarily, to be located with respect to an environment. If that is gone, nothing remains.

## Conclusion: understanding as a self-organizing process

Imagine what it would be like if all minute-to-minute attempts at understanding of our experienced environment were fully conscious and deliberate. There would be no comfortable feeling of being “at home” in the world. Instead, there would be constant confusion and uncertainty, at worst a deep fear of the immediate future as something we cannot, but must, prepare for. In Being and Time, Heidegger describes the state of humans, Dasein, as Being-in-the-World:

In the projecting of understanding, entities are disclosed in their possibility. The character of the possibility corresponds, on each occasion, with the kind of Being of the entity which is understood. Entities within the world generally are projected upon the world—that is, upon a whole of significance, to whose reference relations concern, as Being-in-the-world, has been tied up in advance. When entities within-the-world are discovered along with the Being of Dasein—that is, when they have come to be understood—we say that they have *meaning [Sinn]*. But that which is understood, taken strictly, is not the meaning but the entity, or alternatively, Being. Meaning is that wherein the intelligibility of something maintains itself (Heidegger, 1927).

In Heidegger's terms, lacking an understanding of the kind of Being of an encountered entity is lacking a knowledge of one's possibility in this novel situation. But awareness of one's possibilities of acting is precisely what makes a situation comfortable; we are “at home” in the world when we know what we can do next. Not knowing that would be a condition of fear and hopelessness; our environment would be meaningless. As the quoted passage implies, the significance of our world has been “set up in advance,” meaning that as we move through time we bring with us the structures through which we can understand novel entities. Thus, finding a structure for interpreting newly-encountered entities is not a constant anxiety-ridden necessity but, we may say, a self-organized process that can guarantee our unbroken comfort in our world. This means that the process must be self-organizing, for otherwise we would have no time for acting upon possibilities, but would be in constant destabilizing fear. The conditions for understanding, the “significance of our world” are essential for the existence of possibilities. If dependent upon our conscious organizing powers, each new present moment would be a new cause for alienation and anxiety. That we are not, as a rule, constantly in that state of extreme anxiety, seems to be strong evidence that understanding is a self-organizing process.

Many of the arguments in Sections (a) through (c) are given fuller treatment in Newton (1996).

## Author contributions

NN is the sole author and responsible for all research in the paper.

### Conflict of interest statement

The author declares that the research was conducted in the absence of any commercial or financial relationships that could be construed as a potential conflict of interest.
